# On the Causes of Epidemical Diseases

**Published:** 1832-04

**Authors:** Francis Adams

**Affiliations:** Surgeon, Banchory Ternan, Aberdeen.


### EPIDEMICAL DISEASES.

*On the Causes of Epidemical Diseases.* By Francis Adams, Esq., Surgeon, Banchory Ternan, Aberdeen.

(Concluded from page 194.)

3d. *On the Material Causes of Epidemics.* These have been subdivided into the external and the internal.

By the *external* causes are meant food and drink of an unwholesome nature. Galen gives a most interesting account of an epidemic which prevailed in his time, and was brought on by the use of improper articles of food during the prevalence of a famine:* he remarks, that it furnished a striking illustration of the effect which unwholesome juices have in engendering diseases. The inhabitants of the country around Rome, having consumed all the grain and pulse which had been left them, were compelled to betake themselves to the sprouts of trees and of shrubs, and to bulbous roots and the roots of wild potherbs. Those who partook of this unwholesome food were seized, according to their previous temperaments, with various kinds of ulcers, some with impetiginous, some with scabious, and others with leprous, ulcers; some likewise were seized with fevers, which superinduced fetid and pungent discharges from the bowels, and ended in tenesmus and dysentery. If blood was let in such cases, it was generally found of an unnatural appearance, from which Galen drew the inference that these diseases were occasioned by improper food, which had been converted into unhealthy blood. To food of an improper nature, and especially to summer fruits, the ancient, and many of the modern authorities attribute cholera and other similar complaints, which are apt to prove epidemical during the hot season. Hippocrates, in his Epidemics, relates the histories of many such cases, and Galen remarks that they all arose from the use of unwholesome food. In the Commentaries of Cæsar, mention is made of a plague at Marseilles, brought on by the use of spoilt pannic and barley during a siege of the place.† I may add, that Paulus Ægineta, Avenzoar, and most of the Greek and Arabian authorities, enumerate unwholesome food and drink among the causes of epidemical diseases. Joseph Brown, in his Practical Treatise on the Plague, mentions improper food among the causes of it. This he illustrates by the example of various nations, who, at a time when the atmosphere is in a state of

* De Rebus Boni et Mali Succi. In another work he gives an account of an epidemic occasioned by the use of vitiated wheat. (De Nat. Hum. Comment. 2.)

† De Bello Civil. lib. ii.

distemperature, bring on ardent fevers by the use of acrid food, such as garlic, onions, and the like. The celebrated Dr. Harris ascribes the plague of Marseilles to the use of improper food.* An epidemical sphacelus, described by Langius, prevailed in Lucerne during the year 1709, having been brought on by eating spurred rye.

By the *internal* causes, are meant the fluids of the human body, which have become so altered as to prove deleterious to bodies in health, when conveyed into them. Galen remarks, that the animal fluids may be so changed and corrupted by disease as to resemble, in quality and power, the most destructive venoms of serpents.† This corruption of the animal fluids may be supposed to be occasioned by obstruction of the excrementitious discharges, plethora, and a constitution of the atmosphere which favors putrefaction, namely, a hot and humid intemperament. In this way it is probable, nay almost certain, that all the phenomena of *contagion* are to be accounted for; the morbific particles which, being transmitted from one animal to another, propagate the disease, being formed in the manner we have stated. There is this marked difference, however, between those morbid poisons which are the result of deranged function and the natural venoms of reptiles, that the latter, when conveyed into the body of a healthy animal, cannot propagate themselves by assimilating its fluids to their nature; whereas, this is the distinguishing characteristic of the former. The variolous matter, if conveyed into the body of a healthy animal, not only excites the disease in it, but so multiplies itself as to become capable of contaminating any indefinite number of other animals which may be brought within the range of its infection; but the venom of the viper or of the rattlesnake, although it proves destructive to another animal, does not multiply in that animal so as to enable it to infect others.

This opinion of a morbid poison being formed in the system, and proving the source of contagion, has been pretty extensively entertained by the modern authorities on medicine. Mercurialis, Baptista Susius, Cardanus, and Fabius Paulinus, hold, with Galen, that an internal virus may be formed in the system from corruption of the food, overheated humours, hereditary qualities. According to Joseph Brown, the plague is a subtle poison, either engendered in the human body or communicated to it from without. Kanold maintains that the pestilential contagion is a

* De Peste.

† De Locis Affect. vi. 5; Epidem. iii. § 7; Theriaca ad Pisones.

poison formed by the putrefactive fermentation operating upon unwholesome food in an insalubrious atmosphere.* Morton considers contagions to be a spirituous matter, of the nature of ferments. Fracastorius also maintains that morbid poisons are the result of the putrefactive fermentation; but, in opposition to him, Valeriola held that they are real poisons, that is to say, original substances, and that putrefaction has nothing to do with their formation.† Professor Hufeland supposes them to be formed by a process of *hyperanimalization*; and this is not an improbable conjecture with regard to the febrile virus which appears to be engendered by an excess of the vital actions. When he infers, however, that the pestilential virus bears a near resemblance to the prussic acid, he makes an assertion which does not seem susceptible of proof, and must be founded upon very distant analogies. It is rare, indeed, that sudden deaths take place in the plague, so as to resemble the effects of the prussic acid. All that we can venture to affirm of morbid poisons, then, is, that they consist of the animal fluids, which have undergone some peculiar internal change; that is to say, an *aliation*, to use an appropriate term invented by the learned Mr. James Harris.‡

We shall conclude this paper with a few brief remarks upon the laws by which contagious diseases are propagated.

§ 1. The following chronic diseases are decidedly contagious: Scabies, Ringworm, Syphilis, Elephantiasis, and Yaws.

§ 2. The following have been held to be sometimes contagious: Scrofula and Phthisis.

§ 3. The following acute diseases are generally held to be contagious: Smallpox, Measles, Scarlet fever, common continued Fever, the Plague, and Hydrophobia.

§ 4. The following have been supposed to be at certain times contagious: Intermittent and Remittent Fevers, Influenza, Hooping cough, Erysipelas, Ophthalmia, Puerperal Fever, Dysentery, Ileus, and Cholera.

§ 1. The diseases enumerated in section 1st can all be propagated by contact and by *fomites*. That the contagion of the itch, whether an acrid secretion or a peculiar species

* V. Mangeti, Bibl. Med. Script. lib. xi.

† Sennertus writes thus concerning the pestilential virus: "Qualis sit specifica veneni pestilentis natura et causa, nemo unquam explicare potuit et profectò talia sunt quæ latent animos temperatos, illudunt curiosis." (De Peste.)

‡ Philosophical Arrangements, p. 16.

of animalcules, as some suppose, may be contracted both by touching a diseased person, or by means of clothes and the like which have been in contact with him, can admit of no dispute, being facts ascertained by general experience. The same may confidently be affirmed of all the varieties of ringworm, described by the ancients under the names of Alopœcia, Ophiasis, Area, and Achores.

That Syphilis may be propagated by touch, is admitted by all; and that it may also be contracted from an infected fomes, is clearly proved by a case related by Mr. JOHN HUNTER. FRACASTORIUS also states that the disease is propagated by a fomes. The Sivvens appears to be a disease nearly allied to syphilis, and its contagious powers are of the same character, or even more active.

The contagious nature of Elephantiasis has been proved by so strong evidence, both in ancient and modern times, that I cannot help being surprised it should have been lately disputed. That the disease may be contracted by touch and by a fomes, is maintained by all the authorities; and some of them even express a suspicion that it is communicable by respiration. The more inveterate forms of leprosy, as all the Arabian writers, and FRACASTORIUS and others among the moderns, remark, are apt to pass into elephantiasis; and hence probably the reason why MOSES directs to treat leprosy as a contagious disease.* After diligently comparing the descriptions of elephantiasis given by the Arabians and the older modern writers on medicine, with the first descriptions of the Morbus Gallicus, I am satisfied that the latter is but a modification of the other. This opinion I had formed before reading SPRENGEL's History of Syphilis.

Drs. BATEMAN and BANCROFT affirm that Frambœsia is never contracted but when the matter is applied to broken skin. Dr. WINTERBOTTOM, however, maintains "that the yaws is communicated in the same way as the venereal disease or the itch," that is to say, by contact and by fomites. Dr. JOSEPH ADAMS remarks, that the yaws bears a considerable resemblance to the sivvens of Scotland.† Mr. BENJ. BELL held it to be a variety of the venereal disease.‡

§ 2. Neither Consumption nor Scrofula are generally held by the profession in Britain at the present day to be communicable by any possible means, and yet, with respect to the former of these, the evidence of a contagious nature is very

* Leviticus, chap. xiii.

† On Morbid Poisons, p. 58.

‡ On the Lues Venerea, vol. ii. p. 442.

strong. In the first place, GALEN, RHASES, and all the principal Greek and Arabian physicians, looked upon the disease as being undoubtedly contagious. From the following passage in the works of SALLUST, it would appear that, in the Augustan age, consumption was reckoned synonymous with contagion: "Tanta vis morbi, *uti tabes*, plerosque civium animos invaserat."* Among the modern authorities who maintain this opinion, I may enumerate FRACASTORIUS, SCHENKIUS, ANDRE DU LAURENS, RIVERIUS, HOFFMAN, MORTON, DIEMERBROECK, DESAULT, DARWIN, and Dr. WINTERBOTTOM. The last-mentioned author expresses himself on this subject in the following terms:

"In the list of contagious diseases, we find Phthisis pulmonalis, though not generally acknowledged as such in this country, yet we meet occasionally with such melancholy instances of the disease following close attendance upon a near relative or friend, even in those not generally disposed to it, as must excite considerable apprehension on the subject. No prudent physician would allow a young and healthy person to sleep in the same room with a patient in the advanced stage of phthisis, where it can be avoided; and certainly he would not permit the same bed to be used at any period of the disease."†

I may add, that in Italy, and in America, the profession holds the disease to be contagious. It is to be remarked, however, that this character is not applied to every chronic disease of the thoracic viscera, but merely to consumption properly so called, consisting of scrofulous tubercles in the lungs, in a state of ulceration, and attended with fetid sputa.‡

In the days of Henry the IVth of France, the more inveterate forms of scrofula were supposed to be contagious, both by respiration and by fomites, as will appear from the following certificate given by the Medical School of Paris; "Die vigesima octava Novembris, anno millesimo quingentesimo septuagesimo octavo, Sententia denominatorum virorum de strumis recitata est et approbata. Hæc autem fuit ejusmodi. Supremus Senatus petiit ab ordine Medicorum, An strumæ panificium inficere possent. Respondet, ex mul-

* Catalin. c. 36.

† Edinburgh Med. and Surg. Journal, No. 97.

‡ Dr. RUSH gives an account of a contagious consumption which spread from the proprietors of an estate among the negroes, who were neither related to the first victims nor had been subjected to fatigue or anxiety on their account, and amongst whom it scarcely ever makes its appearance. (Med. Surg. and Obs. &c. vol. i. 8vo. Phil. 1789. Quoted by Dr. MASON GOOD; Study of Medicine, vol. iii. p. 267.)—EDITOR.

torum strumis, ulceribus malignis, virulentis et sordidis laborantium, et eodem in loco una commorantium halitu, panificium infici posse." FRACASTORIUS* and ANDRÉ DU LAURENS† also pronounce confidently that scrofulous ulcers are sometimes contagious.

§ 3. That Smallpox and Measles are contagious, has never, I believe, been disputed from the days of RHASES, who first gave an accurate description of them. The only question which can arise upon this point is, whether or not they may arise in any instance spontaneously, without exposure to contagion. From certain isolated cases which I have observed, under circumstances which seemed to preclude the possibility of infection, I would be inclined to believe in a spontaneous generation. They are certainly communicable by respiration and *per fomitem:* but whether also by touch, is not so easily determined.

All these observations apply also to Scarlatina, which, I am inclined to think, is propagated both as an epidemic and as a contagious disease. I have seen abundant proofs in my own practice, that a person may be capable of communicating the contagion after he has apparently recovered from the fever. With scarlatina we may join Diphtherite and ulcerous Sore-throat, which are nearly allied to it, and obey the same laws of propagation.

The common Continued Fever, called Synochus by the Greeks, Febris Continens by the translators of the Arabian and by the older modern authors, and now generally called Typhus, would certainly appear to me to be contagious in all its forms; and yet the Greeks held that the milder forms of this fever are not contagious; and the same opinion is maintained by the M'Leanites of the present day. (See the Westminster Review, No. vi.)

From THUCYDIDES downwards, all the great writers of antiquity, as far as I can recollect, with the exception of PROCOPIUS, admit the contagious nature of the Plague. I believe, but am not quite certain, that CHIRAC, the professor of medicine at Montpellier, was the first modern physician who denied that the plague is contagious;‡ but of late Dr. MACLEAN has advocated this opinion with singular zeal, and made many proselytes to it: yet I cannot see that the negative experience of a few individuals can be held sufficient to overturn the positive evidence of intelligent men in all ages

* De Contagione. † De Strumarum Natura.

‡ I am aware that PETRAS SALIUS DIVERSAS, who lived about the middle of the sixteenth century, maintained that certain pestilential epidemics are not contagious; but he did not positively deny that the plague is ever contagious.

respecting a matter of fact so open to common observation. So universal was the belief in pestilential contagion in ancient times, that, in the version of the Septuagint, the term plague is used as synonymous with contagion; and ISIDORUS HISPALENSIS says "pestilentia est contagium."* To me it appears that the mass of evidence collected by Dr. RUSSEL to prove that the plagues of Marseilles and Aleppo were contagious, is perfectly unanswerable: indeed, since the more sensible M'Leanists allow that, under particular circumstances, the plague is *contaminative*, they must be considered as almost admitting that mode of communication upon which the contagionists lay most stress, namely, that by respiration. The evidence contained in the works of FRACASTORIUS, PAULINUS, RUSSEL, and MEAD, to prove propagation *per fomitem*, is also very strong. Of contagion by touch, the proofs are not so conclusive, and much negative evidence is produced on the opposite side. The circumstance of Bonaparte's touching with impunity the bodies of persons lying ill of the plague in Cairo, is attested by Dr. ASSALINI and other credible witnesses. That the bodies of persons who have died of the plague are infectious, is denied by FRACASTORIUS and RONDELATIUS; but is confidently affirmed by ZACUTUS LUSITANUS, JOUBERTUS, and DIEMERBROECK.

It is a probable conjecture, made by ALEXANDER APHRODISEIUS,† and adopted by Dr. MEAD, that the hydrophobic virus is formed in the dog during a species of fever. We may take it for granted, I suppose, that the poison is commonly the saliva of the dog in a peculiarly vitiated state. According to CÆLIUS AURELIANUS, ARETÆUS, and VEGETIUS, the disease may be propagated not only by the bite of a rabid dog, but also by his breath; and this fact is confirmed by modern authors, as GOKEL, LISTER, RHAZOUZ, and others. Cælius Aurelianus relates the case of a sewer, who contracted the disease from having sewed a robe which had been torn by a mad dog; and similar cases are related by HILDANUS and HEISTER. We may therefore conclude that the disease is communicable by contact, by respiration, and by a fomes. Cælius Aurelianus further mentions, that the disease may arise spontaneously in the human subject, without any manifest cause; and this fact also is confirmed by modern authority. I myself once saw a case of phrenitis in which the *horror of water* was a symptom.

§ 4. Intermittent and Remittent Fevers are not generally supposed to be communicable by contagion, and yet Dr.

* Origines. † Problem i. 76.

Winterbottom has been assured by physicians, in whom he could place the strongest reliance, that they had known the former of these to be possessed of a contagious character. I can further affirm, that an autumnal remittent fever, which I met with in the year 1818, was decidedly contagious. With regard to the Yellow Fever, I dare scarcely venture to pronounce an opinion, the evidence on both sides so nearly balancing one another; but from what I have seen of febrile diseases in general, I must say that I suspect them all.

Influenza and Hooping cough are often epidemical, but are they not also communicable by contagion? Dr. Cullen refers to certain epidemical catarrhs, which were probably contagious. It is a presumption in favor of this opinion, that the catarrh of horses is admitted to be decidedly contagious. Of the spontaneous generation of hooping cough I once had a proof, which appeared to me irresistible.

That Erysipelas sometimes assumes a contagious character, is rendered pretty evident from the statements contained in Bateman's work on Cutaneous Diseases, and Cooper's Surgical Dictionary. In the year 1823, I met with several cases of erysipelas in the hand which terminated in mortification, and I had strong reason to suspect that the disease was communicable by touch.

The Greek, Latin, and Arabian authorities generally held Ophthalmia to be a contagious distemper. That the purulent ophthalmia brought to this country from Egypt by the British army, about thirty years ago, was contagious, is clearly proved in the works of Vetch, M'Grigor, and Ware: these authors seem to have been of opinion that the disease could only be propagated by the matter being brought to sound eyes by means of clothes, flies, and some such medium.

Puerperal Fever appears to be Synochus complicated with inflammation of the peritoneum. From what I have seen of it, I am satisfied that the abdominal inflammation is symptomatic of the fever, and not the fever of the inflammation. I have also strong reason for suspecting that it may be transmitted from one person to another in the puerperal state, by a third person, who himself remains unaffected. The late Dr. Gordon, of Aberdeen, brought the fullest evidence to prove that in this manner accoucheurs, midwives, and sick nurses, are often the vehicles of the contagion. Mr. Hey, of Leeds, inclines to the same opinion, but is not quite so decided.

Frequent mention is made, both by ancient and modern authors, of the epidemical Dysentery having put on a pestilential character. Many of the cases related in the Epidemics of HIPPOCRATES are of this nature; and GALEN, in his Commentary, mentions that the long-protracted pestilence, which prevailed in his time, proved fatal by superinducing dysenteric discharges from the bowels. FERNEL relates that, in the year 1508, all Europe was desolated by a pestilential dysentery; and the same epidemic is made mention of by PALVERIUS. Epidemical dysenteries, of a malignant nature, are also described by SCHENKIUS and GEORGIUS TURONENSIS. Since the older writers use the term pestilential as nearly synonymous with contagious, it is to be presumed that all these epidemics partook of the contagious nature. The contagious nature of dysentery is so fully established by the evidence of Sir JOHN PRINGLE, Dr. DONALD MONRO, and other physicians of that period, that I can see no good grounds for scepticism upon this circumstance. I agree with SYDENHAM and PRINGLE, that dysentery "is a fever of its own kind, cast inwardly upon the guts:" it is, therefore, essentially different from idiopathic inflammation of the mucous membrane of the intestines.

Though Ileus and Colic are not, in general, either epidemical or contagious diseases, there is strong evidence for believing that they have sometimes assumed these characters. The colic which spread from person to person, and from province to province of the Roman empire, in the days of PAULUS ÆGINETA, is described as contagious by him and AVICENNA. FABIUS PAULINUS, upon the authority of MOYSES ALATINUS, relates that a contagious epidemic of a similar kind prevailed in Mantua, during the year 1560.

All the other causes of epidemical disorders detailed by us in this essay, will be found insufficient to account for the spreading of the epidemical Cholera of the present time, without supposing it to be possessed of a contagious character: for what other cause in the heavens, the air, the waters, and the earth, or what peculiarity in the aliment of mankind, will explain the steady progress which it has been making to the west since its first appearance at Jessore in the year 1817? Since, as we have endeavoured to shew, peritonitis, dysentery, ileus, and colic, have been sometimes met with as contagious diseases, there seems no good reason, *a priori*, to question the possibility of cholera putting on a similar character. This conjecture is rendered more probable by the consideration that the plague is reported to have frequently assumed an aspect little different from that of the spasmodical cholera of the present day. The plague of Athens, as described by THUCYDIDES, is said to have been marked by discharges from the bowels, both upwards and downwards, anxiety, strong contractions; the surface of the body being of a dark, leaden, or pale colour,* or covered with dark-red spots. The expression of countenance and general appearance of a person sinking under this malady, as they are described by LUCRETIUS, bear a wonderful resemblance to the symptoms of cholera as detailed by Sir HENRY HALFORD.†

In membris vero nervi trahier, tremere artus
A pedibusque minutatem succedere frigus
Non dubitabat: item ad supremum denique tempus
Compressæ nares, nasi primoris acumen
Tenue, cavati oculi, cava tempora, frigida pellis,
Duraque, inhorrebat rictum, frons tenta minebat.

Multaque præterea mortis tum signe dabantur,
Perturbata animi mens in mærore, metuque,
Triste supercilium, furiosus vultus et acer;
Sollicitæ porro plenæque sonoribus aures,
Creber spiritus, aut ingens, raroque coortus,
Sudorisque madens per collum splendidus humos.

HIPPOCRATES, who relates many cases of the Athenian epidemic, frequently mentions vomiting as a symptom of them. GALEN also relates that vomiting is the inseparable attendant of the plague, and that looseness of the bowels is a most fatal symptom. In the description of the plague given by RUFFUS, and quoted by PAULUS ÆGINETA, the most striking symptoms are, vomitings of bile, stretching of the hypochondria, pains, coldness of the extremities, discharges of bile from the bowels, and violent contractions. This is evidently a sort of pestilential cholera. The descriptions of the plague by the Arabian authors are so similar, that they may justly be suspected of having copied from Ruffus. Among the symptoms enumerated by RHASES, I remark vomiting and diarrhœa, pain and distention of the belly, coldness of the extremities, and spasmodic contractions. HALY ABBAS mentions vomiting, discharges from the bowels of a bilious, or black nature, or thin, or furfuraceous, with pain

* I have closely followed the interpretation of the scholiast.

† The following is part of Sir Henry Halford's description: "Vomiting or purging, or both these evacuations, of a liquid like ricewater, barleywater, or whey, come on; the features become sharp and contracted; the eye sinks, the look is expressive of terror and wildness; the lips, face, neck, hands, and feet, and soon after the thighs, arms, and whole surface assume a leaden, blue, purple, black, or deep-brown tint. Sometimes there are rigid spasms of the legs, thighs, and loins; the skin is deadly cold, and often damp; the voice is nearly gone; the respiration quick, irregular, and imperfectly performed."

and profuse sweats. The account by Avicenna is little different. So, likewise, in modern times we meet with pestilential epidemics, which are described as having put on the appearance of cholera. The following is part of Ingrassias' description of a plague: "Lor sopraviene ansieta et inquietudine (diciamo volgarmente gran Basca), difficulta di anhelito, con aggiongersi subito, il vomito, nausea, con prostration di appetito, over con dolor di stomacho or certo mordication di quello, che chiamano i Medici *morsum cordis*." The appearance of the vomitings are more minutely given in another place: "Similmente il vomito non solamente suole essere de flegma, et piu diverse sorti de cholere verdi chiamate dai medici prassine, eruginose, vitelline, rosse, chialle, citrine, et miste, quel che è peggio spesse volte puzzolenti, ed alle volte mescolate con vermi vivi et piu delle volte morte." In the plague of Aleppo, during the years 1760, 1761, and 1762, as minutely described by Dr. Russel, one class of cases was distinguished by violent vomiting, anxiety, burning pain about the præcordia, loss of speech, looseness of the bowels, convulsive motions of the limbs, disturbed aspect, and broad livid patches upon the surface of the body: they were unattended by carbuncles and buboes, and often proved fatal in twenty-four hours. These cases evidently resembled the pestilential cholera now prevailing in the west of Europe. From Diemerbroeck's description of the plague at Nimeguen, in the year 1636, it appears that the worst cases were generally attended with vomiting, singultus, and looseness of the bowels, and discoloration of the skin, anxiety, internal heat, spasmodic contraction of the limbs, and expression of terror in the countenance. Delirium was a rare symptom. According to Hodges, Harris, and Sydenham, nausea, pain in the region of the stomach, violent vomitings, and purple spots were the inseparable concomitants of the plague of London, in 1665.

But my limits at present will not permit me to enter further into the modern history of the plague. Enough, however, has been said to shew that it is by no means uncommon for the plague to simulate the appearances of idiopathic cholera. That the fatal epidemic which has desolated the East, and, it is to be feared, has now reached England, partakes of the pestilential character, seems but too probable; although, as I have never seen it, and can judge of it only from the reports of others, it becomes me not to pass too confident an opinion regarding its nature. But, before concluding, I would beg leave to recommend to those members of the profession who have acquired a practical acquaintance with this epidemic, to compare their own experience with that of former times, as being the most likely way of throwing some additional light upon a distemper which has been so long unknown in this country.

